# Supplementary material for: Aluminum foil negative electrodes with multiphase microstructure for all-solid-state Li-ion batteries
Source: Nat Commun. 2023 Jul 18;14:3975. doi: 10.1038/s41467-023-39685-x (PMC10354103; doi:10.1038/s41467-023-39685-x)
Supplement: Supplementary file 1 — Supplementary information [file 41467_2023_39685_MOESM1_ESM.pdf]

Supplementary Information

Aluminum Foil Negative Electrodes with Multiphase Microstructure for All-Solid-State Li-Ion Batteries

Yuhgene Liu[1], Congcheng Wang[2], Sun Geun Yoon[2], Sang Yun Han[2], John A. Lewis[1], Dhruv Prakash[1], Emily J. Klein[1], Timothy Chen[2], Dae Hoon Kang[3], Diptarka Majumdar[3], Rajesh Gopalaswamy[3], Matthew T. McDowell[1,2]*

[1]School of Materials Science and Engineering, Georgia Institute of Technology, Atlanta, GA, 30332, USA.

[2]George W. Woodruff School of Mechanical Engineering, Georgia Institute of Technology, Atlanta, GA, 30332, USA.

[3]Novelis, Inc., Kennesaw, GA 30144, USA.

*Corresponding Author: mattmcdowell@gatech.edu

SUPPLEMENTARY FIGURES

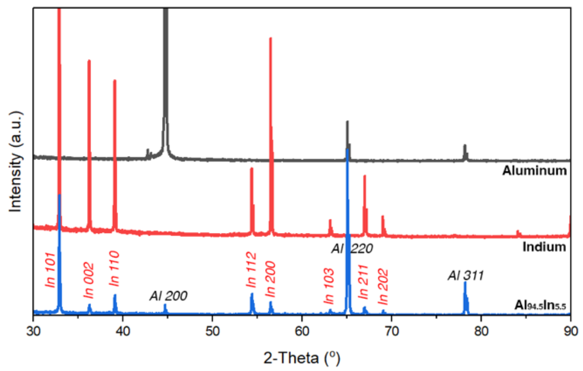

Supplementary Figure 1. X-ray diffraction (XRD) spectra of pristine aluminum (ICDD 04-012-7848), indium (ICDD 01-808-5363), and $Al_{94.5}In_{5.5}$ alloy.

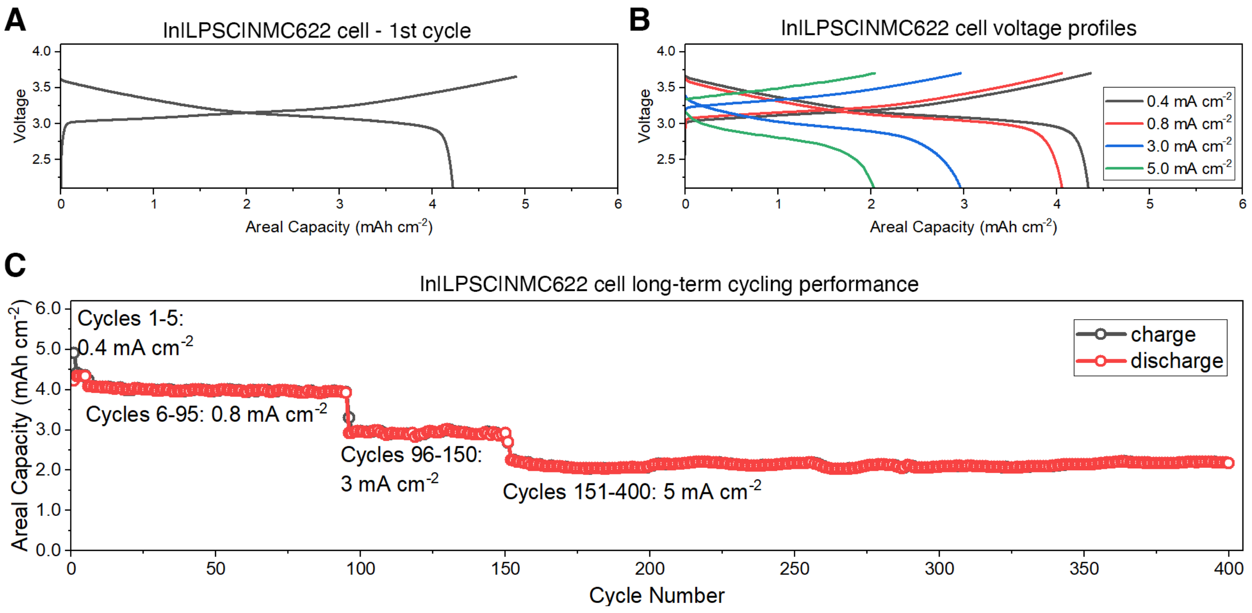

Supplementary Figure 2. Galvanostatic cycling of a full SSB cell with a 30-μm thick indium foil electrode (5.8 mAh cm^{-2} of NMC622 loading and 24 MPa stack pressure). The cell was charged to a voltage cutoff of 3.7 V and discharged to 2.1 V. The initial five cycles were run with the lower current density of 0.4 mA cm^{-2} before ramping to 0.8 mA cm^{-2} for cycles 6 to 95, 3 mA cm^{-2} for cycles 96 to 150, and 5 mA cm^{-2} for subsequent cycles. (A) First-cycle voltage curve, showing 86% initial CE. (B) Voltage curves under different current densities throughout cycling. (C) Areal capacity over 400 cycles. Cell testing was performed at 25 °C.

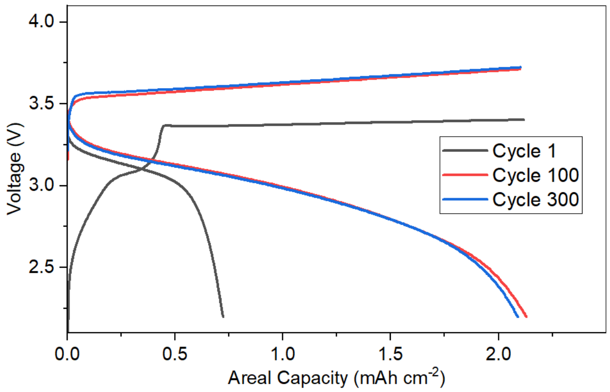

Supplementary Figure 3. Voltage curves from galvanostatic testing of an $Al_{94.5}In_{5.5}$|LPSC|NMC622 cell at 0.5 mA cm^{-2} for the first cycle and 2.0 mA cm^{-2} for the subsequent cycles under constant-capacity testing conditions (lithiation capacity controlled to be 2.1 mAh cm^{-2}); these curves correspond to the cycling data in Fig. 3A. This cell has a significant excess of NMC (16 mAh cm^{-2}), and it was tested under 50 MPa stack pressure at 25 °C.

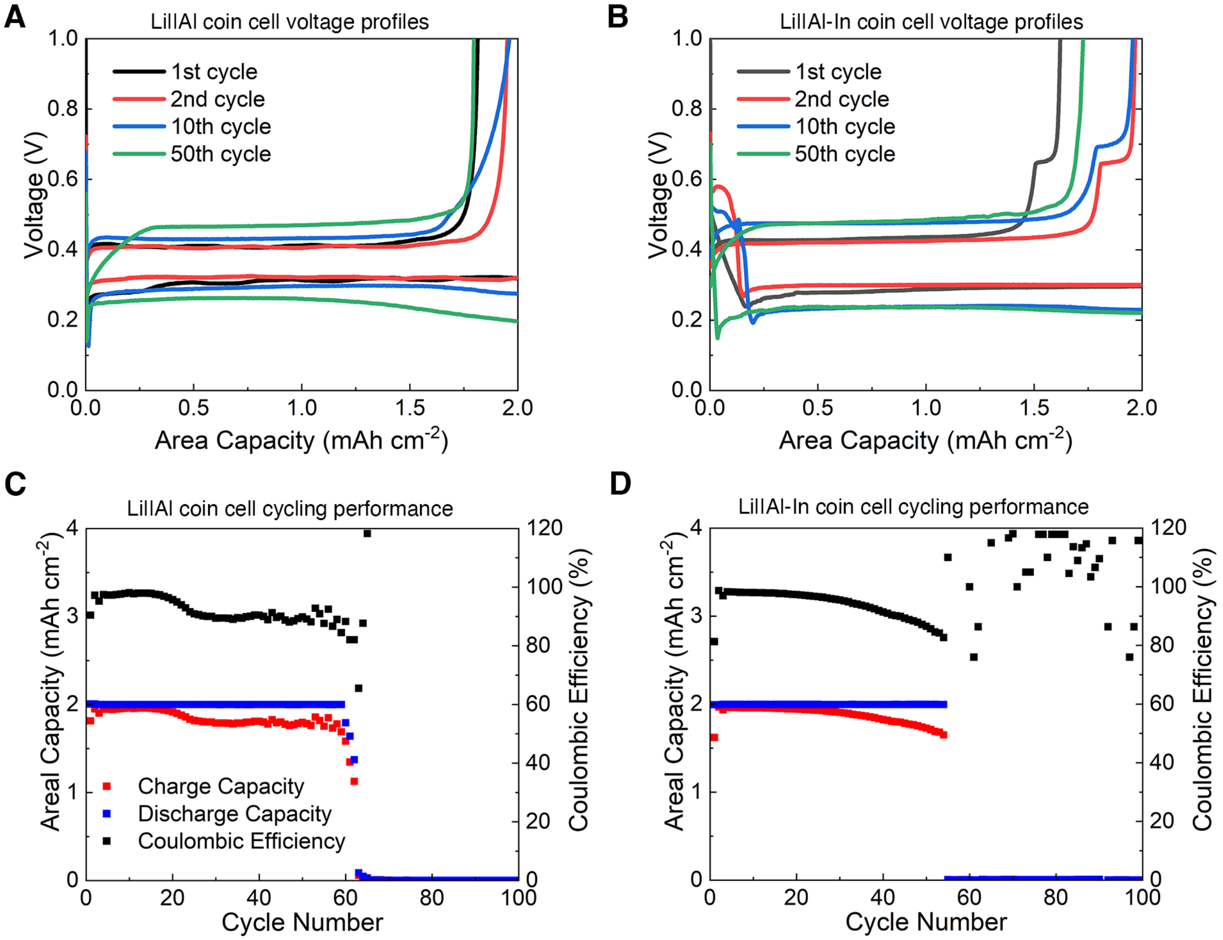

Supplementary Figure 4. Galvanostatic cycling of Li||Al-based coin cells using 1.0 M $LiPF_6$ in EC:DEC (1:1 by volume) with 10 vol% FEC electrolyte solution at 1 mA cm^{-2} current density. (A) Pure Al foil voltage curves. (B) Al-In (5.5 at% In) foil voltage curves. (C) Pure Al foil

capacity and CE plot. (D) Al-In (5.5 at% In) capacity and CE plot. All testing was performed at 25 °C.

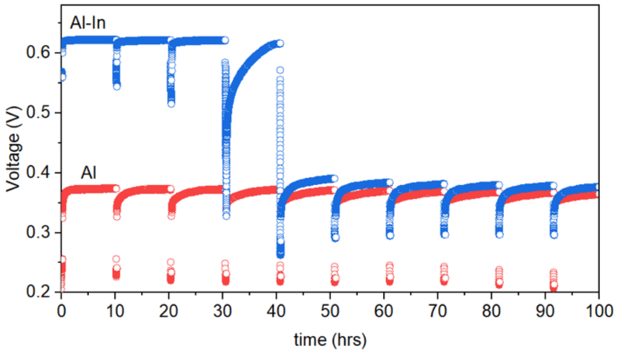

Supplementary Figure 5. Galvanostatic intermittent titration technique (GITT) experiments on aluminum and Al-In cells with lithium metal counter electrodes at 10 MPa. Data showing voltage traces with time for 10-min 0.4 mA pulses and 10-h rest periods for aluminum (red) and Al-In (blue). Testing was performed at 25 °C.

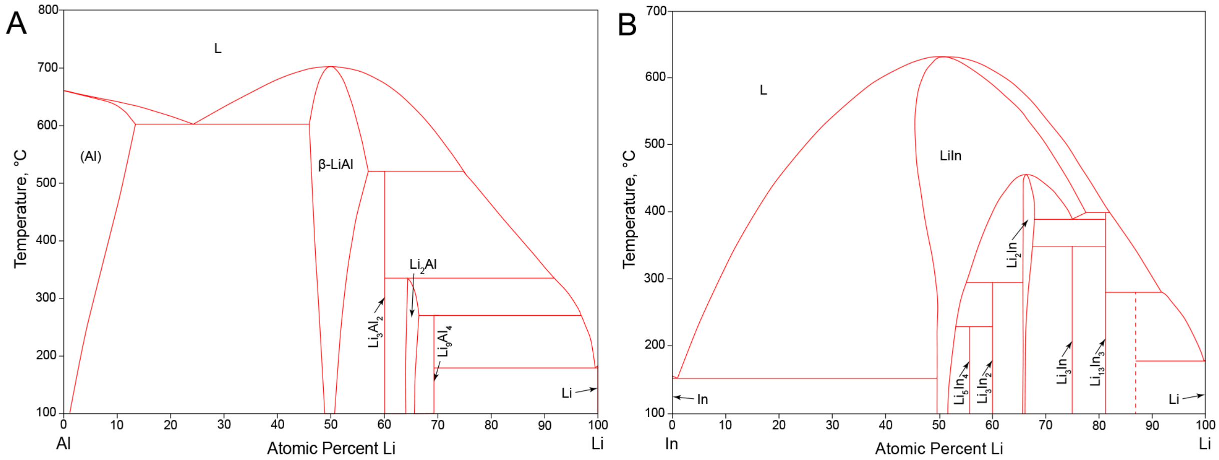

Supplementary Figure 6. Binary phase diagrams for (A) Al and Li; (B) In and Li. Panel (A) is modified from data in supplementary ref. 1, and panel (B) is modified from data in supplementary ref. 2.

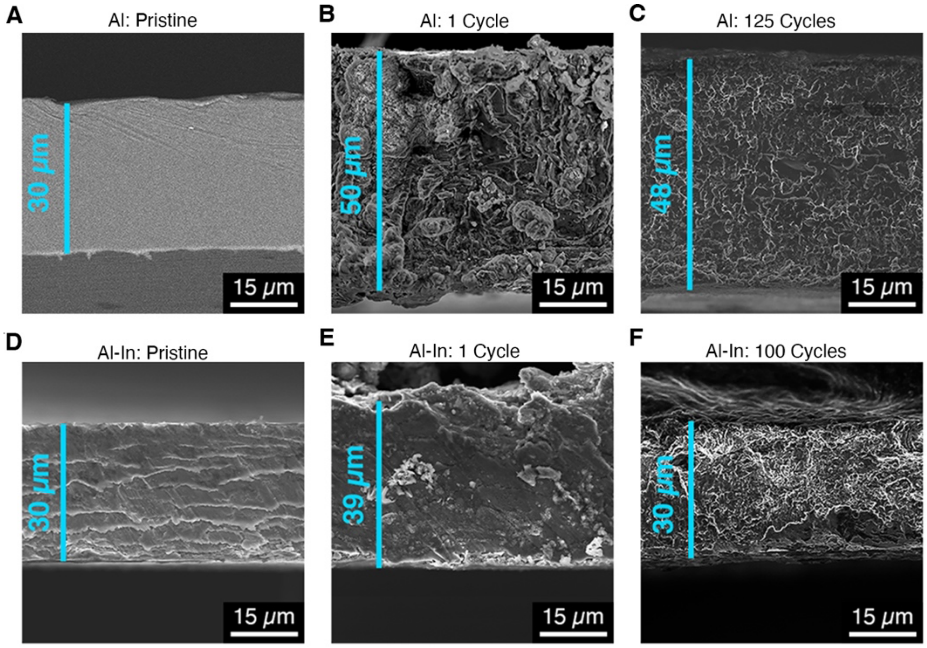

Supplementary Figure 7. Cross-sectional SEM images of Al-based negative electrodes at different stages of cycling, in which the samples were prepared by manual cutting with a sample, which obscures some microstructural features. (A) Pristine aluminum foil, **(B)** aluminum foil after one cycle, and **(C)** aluminum foil after 125 cycles. The slight thickness increase with cycling is likely due to internal cracks and porosity. **(D)** Pristine $Al_{94.5}In_{5.5}$ foil, **(E)** $Al_{94.5}In_{5.5}$ foil after one cycle, and **(F)** $Al_{94.5}In_{5.5}$ foil after 100 cycles. All cells for SEM were assembled with stack pressures of 24 MPa and cycled at 0.8 mA cm^{-2} with positive electrode loadings of 5.8 mAh cm^{-2}. All foils are shown in the discharged (delithiated) state. Testing was performed at 25 °C.

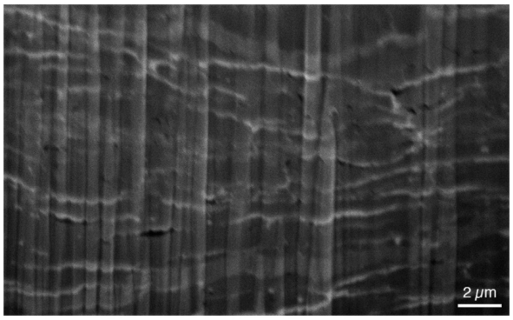

Supplementary Figure 8. Cryogenic-FIB-SEM of an $Al_{94.5}In_{5.5}$ electrode after 20 full lithiation/delithiation cycles in a cell with an NMC positive electrode. The cell was assembled with a stack pressure of 50 MPa and cycled at 0.7 mA cm^{-2} with positive electrode loading of 3.0 mAh cm^{-2}, with the pristine foil being 11 μm thick. The morphology of the surface is due to curtaining during FIB milling. Testing was performed at 25 °C.

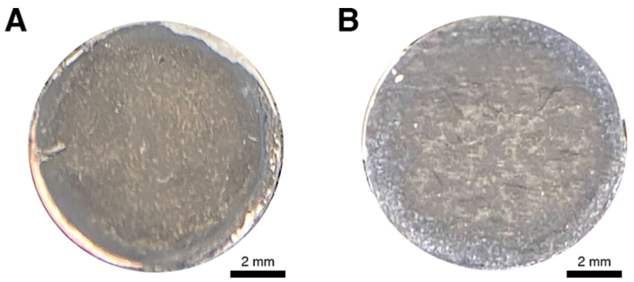

Supplementary Figure 9. Photographic pictures of **(A)** Aluminum electrode after 125 cycles and **(B)** Al-In electrode after 100 cycles in a SSB. Testing was performed at 25 °C with a stack pressure of 24 MPa. Cycles 1-2 were performed at 0.2 mA cm^{-2}, cycles 3-5 were performed at 0.4 mA cm^{-2}, and the remaining cycles were performed at 0.8 mA cm^{-2}.

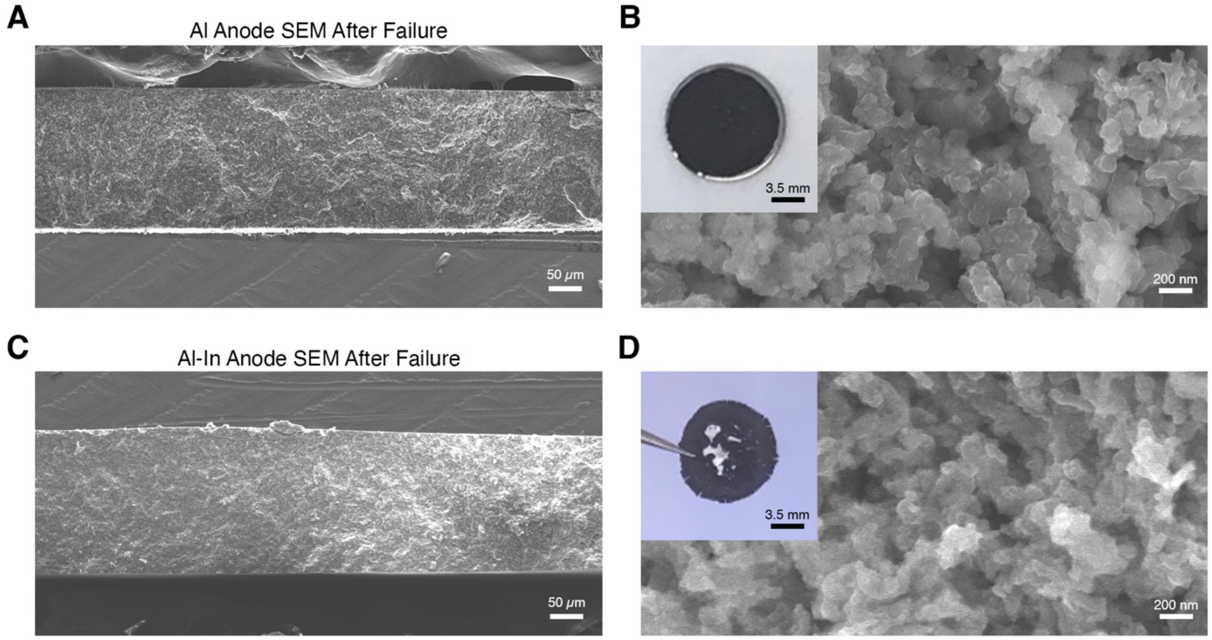

Supplementary Figure 10. SEM images of Al-based electrodes tested in coin cell configurations using Li metal as counter electrode and 1.0 M $LiPF_6$ in EC:DEC (1:1 by volume) with 10 vol% FEC electrolyte solution. The initial thickness of the Al-based electrodes was 30 μm. **(A)** Cross-sectional image of aluminum after failure when cycling to 59 cycles at 1 mA cm^{-2}. **(B)** Magnified image showing porosity within the aluminum foil, along with a top-down photograph of the electrode. **(C)** Al-In (5.5 at% In) after failure when cycling to 54 cycles at 1 mA cm^{-2}. **(D)** Magnified image showing porosity within the Al-In foil, along with a top-down photograph of the electrode. Testing was performed at 25 °C.

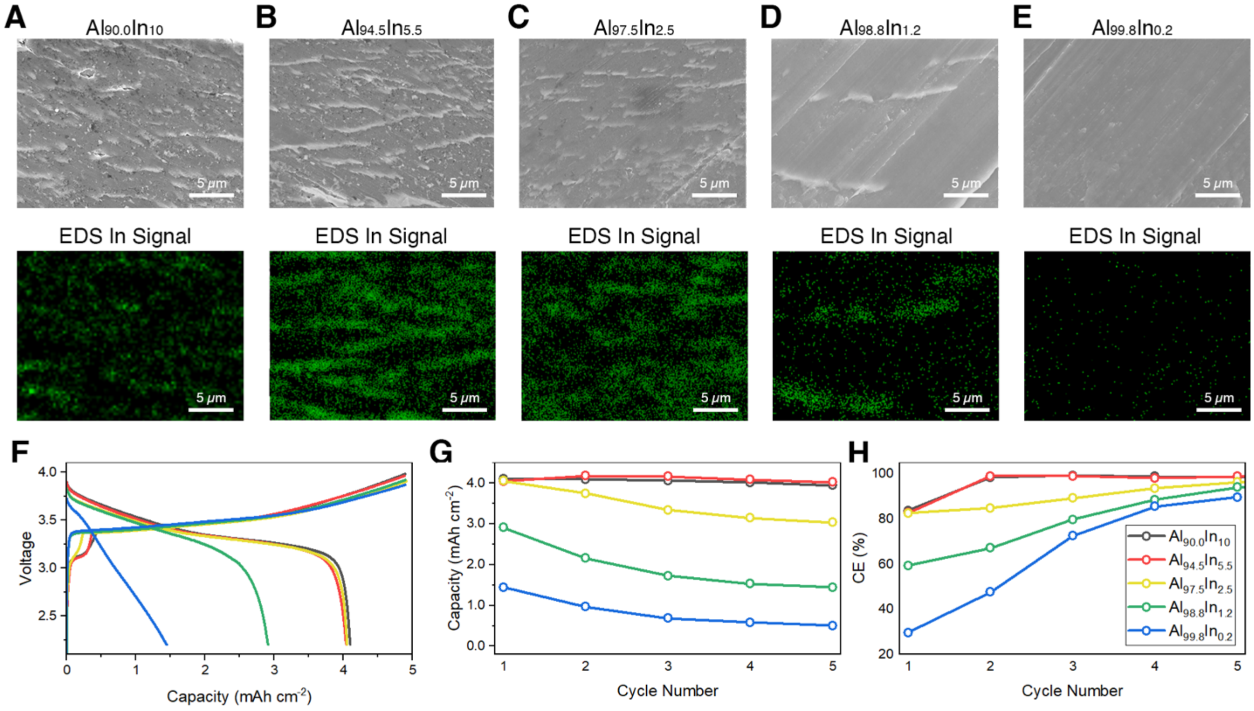

Supplementary Figure 11. Microstructure and electrochemical behavior of Al-based electrodes with different In content. **(A-E)** Cross-sectional SEM image (top) and indium EDS signal (bottom) from different foils: **(A)** $Al_{90}In_{10}$, **(B)** $Al_{94.5}In_{5.5}$, **(C)** $Al_{97.5}In_{2.5}$, **(D)** $Al_{98.8}In_{1.2}$, and **(E)** $Al_{99.8}In_{0.2}$. **(F)** Voltage curves of the first cycle of cells with 30-µm thick Al-In electrodes and 5.8 mAh cm^{-2} NMC622 loading. The In content in each alloy was different according to the legend in (H). Cells were cycled at 0.8 mA cm^{-2} and had a stack pressure of 50 MPa. **(G)** Areal capacity over the first few cycles of the cells in (F). **(H)** Coulombic efficiency over the first few cycles of the cells in (F). Testing was performed at 25 °C.

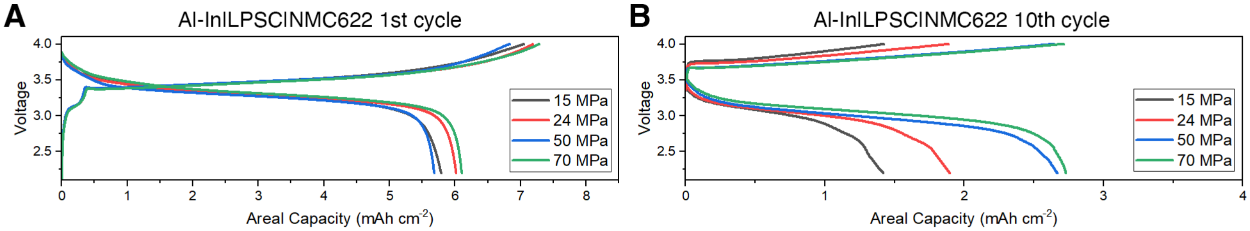

Supplementary Figure 12. Investigating of effects of stack pressure on full cells with $Al_{94.5}In_{5.5}$ electrodes with 8.3 mAh cm^{-2} of NMC622 loading. **(A)** Voltage curves of the first cycle under stack pressures of 15, 24, 50, and 70 MPa using a current density of 0.8 mA cm^{-2}. **(B)** Voltage curves of the 10th cycle under a current density of 6.5 mA cm^{-2}. Testing was performed at 25 °C.

Supplementary Table 1. Comparison of cycling performance of ASSBs presented in this work to other literature reports.

Working Electrode	Count-er Elect-rode	Working Electrode Thick-ness (µm)	Working Electrode Pre-Lithiation (YES/NO)	Cap. (mAh cm^{-2})	Current Density (mA cm^{-2})	Avg. Cell Disch. Volt. (V)	Cycles	Avg. CE (%)	Cap. Ret. (%)	Stack Press. (MPa)	T (°C)	SSE	Ref.
Li/Ag-C	NMC 90:5:5	5 - 10	No	4.6	3.4	~3.7	1000	99.8	89	2	60	Li_6PS_5Cl	3
Porous Si	Li	4.7	No	2.2	0.1	~0.4	100	99.8	93	120	25	$80Li_2S$-$20P_2S_5$	4
Micro-Si	NMC 811	12	No	2.0	5	~3.5	500	99.9	80	50	25	Li_6PS_5Cl	5
In-Li1%	NMC 811	50	Yes	4.0	4.8	~3.7	740	99.98	92	760	25	$Li_{10}Si_{0.3}PS_{6.7}Cl_{1.8}$	6
$Li_{0.8}Al$	S	-	Yes	1.3	0.35	~1.5	200	99.96	93	300	25	$Li_{10}GeP_2S_{12}$	7
Al	LCO	100	Yes	2.2	0.72	~3.4	300	100	~100	100	25	Li_6PS_5Cl	8
Al-In	NMC 622	30	No	2.3	6.5	~3.5	200	99.7	85	50	25	Li_6PS_5Cl	This work
	NMC 622	30	No	2.1	2	~3.5	500	98.9	100		25	Li_6PS_5Cl	

Supplementary Table 2. Fitted parameters from EIS.

	R1		Q1		R2		Chi-Square
	Ohm cm^2		F s^{a-1}		Ohm cm^2		
**Al**	22.3	± 1.8	97.7×10^{-6}	$\pm 0.12 \times 10^{-3}$	23.5	± 4.9	0.81
**Al-In**	23.2	± 1.9	0.574×10^{-3}	$\pm 1.6 \times 10^{-3}$	10.3	± 4.8	0.028

Supplementary Note 1: Energy Calculations for Figure 1

Specific energy (Wh kg^{-1}) and energy density (Wh L^{-1}) for the various battery configurations in Figure 1 were calculated at the stack level (i.e., without considering external packaging) by considering the separator, negative electrode, positive electrode, and current collectors. All cells assumed an areal discharge capacity of 4 mAh cm^{-2}, a 20-µm separator, a 10-µm copper (8.96 g cm^{-3}) current collector, and a 10-µm aluminum (2.7 g cm^{-3}) current collector.

Li-ion Cell with Non-Aqueous liquid electrolyte solution. The active positive electrode material is NMC 811 with a theoretical discharge capacity of 200 mAh g^{-1} and density of 4.78 g cm^{-3}. The slurry cast positive electrode has 96 wt. % active material, 2% Solef 5130 binder, 2% Imerys C65 conductive additive. The electrode has 30% porosity and a total thickness of 66 µm. The N:P ratio

is 1.1 so the graphite negative electrode would hold 4.4 mAh cm^{-2}. Similar conditions of 96 wt% active material, 2 wt% binder, and 2 wt% conductive additive were applied to the slurry cast negative electrode. The electrode has 32% porosity and a total thickness of 85 μm. The polypropylene separator has a density of 0.92 g cm^{-3} and the electrolyte has a density of 1.3 g cm^{3}. The mean discharge voltage of this cell is 3.7 V.

SSE for SSB. The solid-state electrolyte is Li_6PS_5Cl with a density of 1.86 g cm^{-3}. The SSE is 20-μm thick.

Composite Positive Electrode for SSB. The active material is NMC 811. The composite is 80 wt% NMC 811, 17.5 wt% SSE, and 2.5 wt% conductive carbon. The composite electrode has a total thickness of 69 μm.

1x Excess Lithium Negative Electrode for SSB. The N:P ratio is 1.0 so the lithium metal foil would hold 4.0 mAh cm^{-2}. Lithium's specific capacity is 3860 mAh g^{-1}, and it has a density of 0.534 g cm^{-3}. The negative electrode thickness is 19 μm. The mean discharge voltage of this cell is 3.8 V.

Dense Silicon Negative Electrode for SSB. The N:P ratio is 1.1 so the slurry-cast 99.9% silicon electrode would hold 4.4 mAh cm^{-2}. Silicon's specific capacity is 3579 mAh g^{-1} and it has a density of 2.3 g cm^{-3}. The binder is PTFE and it makes up 0.1 wt% of the slurry cast negative electrode. The electrode thickness is 9 μm. The mean discharge voltage of this cell is 3.4 V.

Aluminum foil Negative Electrode for SSB. The N:P ratio is 1.1 so the aluminum foil electrode would hold 4.4 mAh cm^{-2}. Aluminum's specific capacity is 990 mAh g^{-1} and it has a density of 2.7 g cm^{-3}. The electrode thickness is 16 μm. The mean discharge voltage of this cell is 3.45 V.

Differential Capacity (dQ/dV) Analysis. *dQ/dV* curves were calculated in OriginLab by linearly interpolating the data and taking the first derivative. A Savitzky-Golay smoothing filter was applied to obtain the final curves.

Supplementary References

1. Okamoto, H. Al-Li (aluminum-lithium). *J. Phase Equil. Diffus.* **33**, 500–501 (2012).
2. Songster, J. & Pelton, A. D. The In-Li (indium-lithium) system. *J. Phase Equil.* **12**, 37–41 (1991).
3. Lee Y., Fujiki, S., Jung, C., Suzuki, N., Yashiro, N., Omoda, R., Ko, D., Shiratsuchi, T., Sugimoto, T., Ryu, S., Hwan Ku, J., Watanabe, T., Park, Y., Aihara, Y., Im, D., & Taek Han, I. High-energy long-cycling all-solid-state lithium metal batteries enabled by silver–carbon composite anodes. *Nat. Energy* **5**, 299–308 (2020).
4. Sakabe, J., Ohta, N., Ohnishi, T., Mitsuishi, K., & Takada, K. Porous amorphous silicon film anodes for high-capacity and stable all-solid-state lithium batteries. *Commun. Chem.* **1**, 24 (2018).
5. Tan, D. H. S., Chen, Y., Yang, H., Bao, W., Sreenarayanan, B., Doux, J., Li, W., Lu, B., Ham, S., Sayahpour, B., Scharf, J., Wu, E. A., Deysher, G., Han, H. E., Hah, H. J., Jeong, H., Lee, J. B., Chen, Z., & Meng, Y. S., Carbon-free high-loading silicon anodes enabled by sulfide solid electrolytes. *Science* 373, 1494–1499 (2021).
6. Wang, Z., Zhao, J., Zhang, X., Rong, Z., Tang, Y., Liu, X., Zhu, L., Zhang, L., & Huang, J. Tailoring lithium concentration in alloy anodes for long cycling and high areal capacity in sulfide-based all solid-state batteries. *eScience* **3**, 100087 (2022).
7. Pan, H., Zhang, M., Cheng, Z., Jiang, H., Yang, J., Wang, P., He, P., & Zhou, H. Carbon-free and binder-free Li-Al alloy anode enabling an all-solid-state Li-S battery with high energy and stability. *Sci. Adv.* 8, eabn4372 (2022).
8. Fan, Z., Ding, B., Li, Z., Hu, B., Xu, C., Xu, C., Dou, H., & Zhang, X. Long-cycling all-solid-state batteries achieved by 2D interface between prelithiated aluminum foil anode and sulfide electrolyte. *Small* 2204037 (2022).
